# A LONELY GUY protein of *Bordetella pertussis* with unique features is related to oxidative stress

**DOI:** 10.1038/s41598-019-53171-9

**Published:** 2019-11-19

**Authors:** Filippo Moramarco, Alfredo Pezzicoli, Laura Salvini, Rosanna Leuzzi, Werner Pansegrau, Enrico Balducci

**Affiliations:** 1 GSK Vaccines, Via Fiorentina 1, 53100 Siena, Italy; 2 Department of Pharmacy and Biotechnology (FaBiT), University of Bologna, Via Belmeloro 6, Bologna, 40126 Italy; 3 Toscana Life Sciences Foundation, Via Fiorentina 1, 53100 Siena, Italy; 4 School of Biosciences and Veterinary Medicine, University of Camerino, via Gentile III da Varano, 62032 Camerino, Italy

**Keywords:** Hydrolases, Infection

## Abstract

The Gram-negative bacterium *B. pertussis* is the causative agent of whooping cough. This infection is re-emerging and new features related to Bordetella pathogenesis and microbiology could be relevant to defeat it. Therefore, we focused our attention on BP1253, a predicted exported protein from *B. pertussis* erroneously classified as lysine decarboxylase. We showed that BP1253 shares the highly conserved motif PGGxGTxxE and the key catalytic amino-acid residues with newly structurally characterized “LONELY GUY” (LOG) proteins. Biochemical studies have confirmed that this protein functions as a cytokinin-activating enzyme since it cleaves the N-glycosidic linkage between the base and the ribose, leading to the formation of free bases, which are the active form of plant hormones called cytokinins. Remarkably, BP1253 selectively binds monophosphate nucleotides such as AMP, GMP and CMP, showing a wider variety in binding capacity compared to other LOGs. Cytokinin production studies performed with *B. pertussis* have revealed 6-O-methylguanine to be the physiological product of BP1253 in agreement with the higher activity of the enzyme towards GMP. 6-O-methylguanine is likely to be responsible for the increased sensitivity of *B. pertussis* to oxidative stress. Although BP1253 has a primary sequence resembling the hexameric type-II LOGs, the dimeric state and the presence of specific amino-acids suggests that BP1253 can be classified as a novel type-II LOG. The discovery of a LOG along with its product 6-O-methylguanine in the human pathogen *B. pertussis* may lead to the discovery of unexplored functions of LOGs, broadening their role beyond plants.

## Introduction

*Bordetella pertussis* (Bp) is the human-specific encapsulated Gram-negative bacterium that causes whooping cough, a highly contagious infection of the respiratory tract. The pertussis disease is endemic worldwide, affecting people of all ages, and becoming particularly severe and even fatal in young infants^1^. Despite high vaccination coverage worldwide, the number of pertussis cases is increasing^2–4^. The resurgence of pertussis could be ascribed to different causes: epidemiological changes in the circulating strains^5,6^ and the different immunological response triggered by acellular vaccine (aP) compared to whole cell pertussis vaccine^7^, leading to waning immunity^8^. In addition, the difficulty of diagnosing the disease in the early phase of infection leads to a loss of effectiveness of the antibiotic treatment and, in severe cases, the patients may also not respond to the therapies due to breathing difficulties^1^. The continuous increase in pertussis cases across all age groups is a serious public health issue. To resolve this problem, the scientific community is operating on several fronts: on the one hand, it is trying to improve the current vaccine, prevent transmission and provide a long lasting immunity. On the other hand, scientists are attempting to identify novel virulence factors, which could lead to the discovery of new mechanisms of pathogenesis. This would pave the way to alternative therapeutic agents and target treatments in order to reverse the disease at the time of diagnosis.

In an effort to identify and characterize new proteins involved in Bp pathogenesis, we focused our attention on BP1253, a putatively exported protein and potential antigen with an unknown function, previously classified as lysine decarboxylase (LDC).

Cytokinins are plant hormones involved in many important physiological processes, such as growth and branching^9^, chloroplast development^10^, delay of leaf senescence^11^ and activation of plant defense responses^12^. LONELY GUY proteins (LOGs) convert inactive cytokinin nucleotides to biologically active cytokinins by means of their phosphoribohydrolase (PRH) activity, which results in the hydrolysis of the N-glycosidic linkage between the base and the ribose^13^. LOGs can be classified into two clusters based on their oligomeric state: type-I LOGs are dimeric while type-II LOGs are hexameric. Type-I and type-II LOGs may be subdivided into two further sub-groups based on variations in amino acids in the substrate binding site^14^.

In this study, combining *in silico* analysis and biochemical characterization, we demonstrate that BP1253 is a homolog of LOG, the plant cytokinin-activating enzyme. The position of BP1253 in the LOG family phylogenetic tree indicates BP1253 as a novel type of LOG possessing PRH activity. Remarkably, while cytokinins are adenine-derived compounds, the physiological product of BP1253 is 6-O-methylguanine, which could represent a member of a new class of molecules with citokinin-like activity. The accumulation of this product is probably responsible for the sensitization of Bp to oxidative stress. Cytokinin-activating homologs of plant LOGs have been recently identified in the human pathogens *Mycobacterium tuberculosis* (MtLOG)^15^ and *Pseudomonas aeruginosa* PAO1 (PaLOG)^16^. Therefore, the discovery of a LOG protein with unique features in Bp suggests that LOGs and cytokinins could play an important role in human pathogens.

## Results

### BP1253 is a homolog of the plant cytokinin-activating enzyme LOG

**Figure 1:**
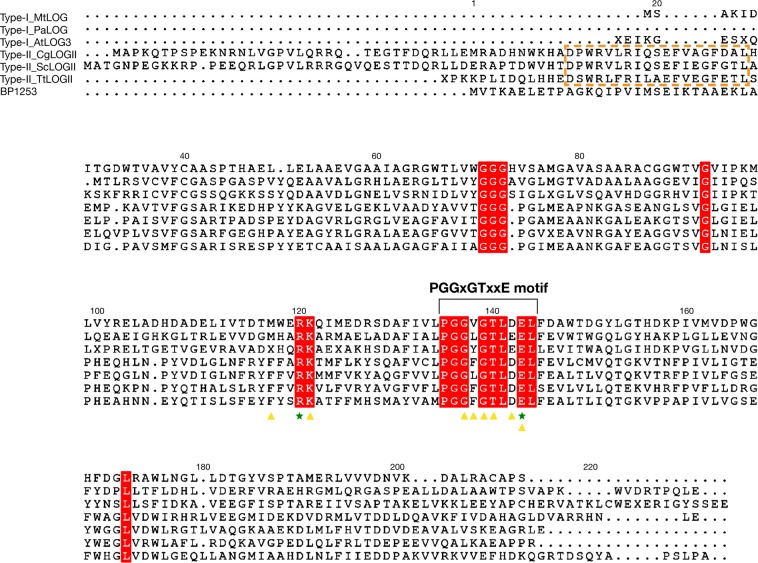
BP1253 from *B. pertussis* is a homolog of LOG proteins. Sequence alignment of type-I and type-II LOG proteins and BP1253 generated by *Clustal omega* and drawn with *ESPript* 3.0 software. Residues involved in the catalysis are indicated with green-colored stars while the amino acids that accommodate AMP in the catalytic site are indicated by yellow-colored triangles. A bracket indicates the PGGxGTxxE motif, while the helix present in other type–II LOGs is indicated with an orange–colored dotted rectangle. The amino acids shared by all LOG proteins are highlighted in red. MtLOG, PaLOG and AtLOG are abbreviations of type-I LOG proteins from *M. tuberculosis*, *P. aeruginosa* and *A. thaliana* respectively. CgLOG, ScLOG and TtLOG indicate type-II LOGs from *C. glutamicum, S. coelicor* and *T. thermophiles*.

**Figure 2:**
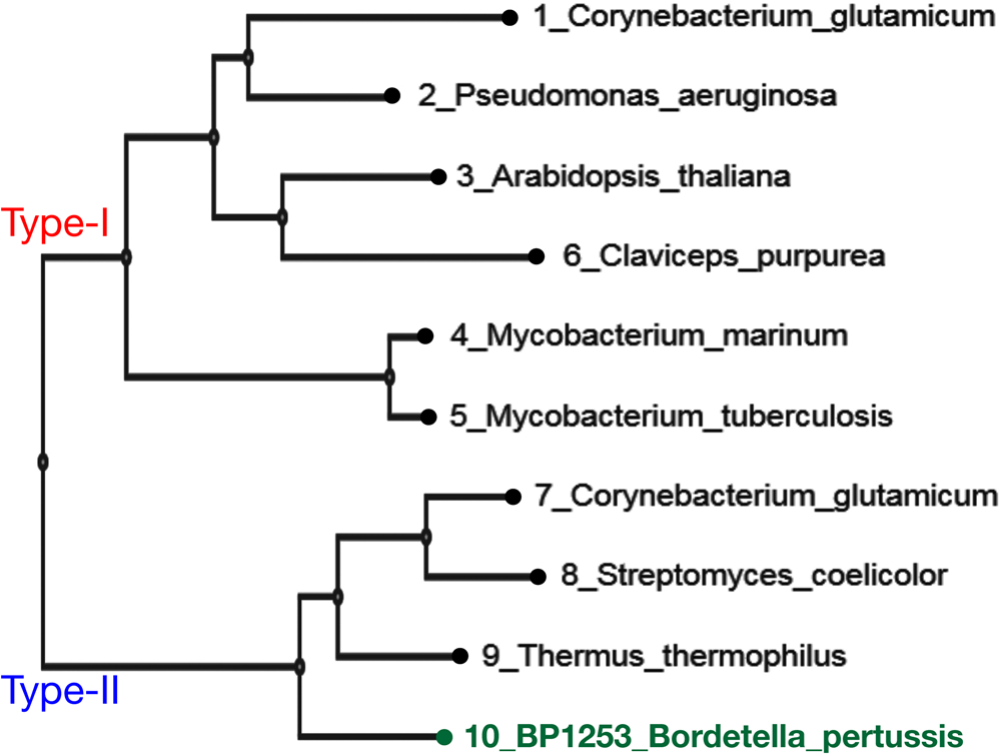
Phylogenetic analyses of LOG-like proteins and *B. pertussis* BP1253. The phylogenetic tree was realized with MAFFT and visualized through the software tool Archaeopteryx.js. BP1253 is highlighted in green. The proteins are divided into two branches: one represents the Type-I LOGs and the other one the Type-II LOGs. The dimeric BP1253 is grouped with Type-II LOGs forming hexamers.

BP1253, along with many proteins from different organisms of various kingdoms, like *Plantae, Animalia, Bacteria* and *Fungi*, was miss-annotated as lysine decarboxylase (LDC)^17^. A more accurate *in silico* analysis has revealed that BP1253 shares with LOG proteins not only the highly conserved motif PGGxGTxxE, but also the Arg and Glu residues of the catalytic core (Fig. 1). BP1253 shows a higher sequence homology with the type-II LOGs, which form hexamers, as compared to type-I LOGs, which form dimers^14,18^. In order to characterize BP1253, we expressed it as a His-tagged recombinant protein (Supplementary Fig. S1). The expression of BP1253 in Tohama I and knock-out Tohama I Δ1253 strain is also shown (Supplementary Fig. S1). The size exclusion chromatography (SEC) of BP1253 showed a main peak of 50 kDa (Supplementary Fig. S2), while the multi-angle light scattering (MALS) analysis showed a protein with a molecular weight of 49.32 (±5%) kDa with a polydispersity of 1.010 (±7.7%; Supplementary Fig. S2). Both methods showed that BP1253 is a dimer. The position of BP1253 in the LOG family phylogenetic tree between proteins classified as type-I or type-II suggests that BP1253 could represent a novel type-II group of LOGs (Fig. 2).

### Nucleotide binding activity of BP1253

**Figure 3:**
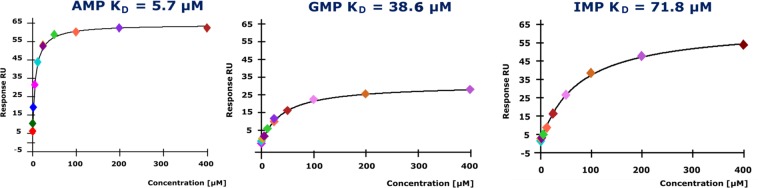
Surface Plasmon Resonance study. Biacore analysis revealed that BP1253 has the ability to bind AMP, GMP and IMP with a dissociation constant of 5.7, 38.6 and 71.8 µM respectively. BP1253-His was immobilized via amine coupling procedure on Sensor chip CM5. Concentrations from 0.78 to 400 μM were used to measure the K_D_. Identical results were obtained by three independent experiments.

To get insight into the ability of BP1253 to bind AMP, which is the substrate of LOGs commonly used in *in vitro* assays, we first resorted to a theoretical approach. This involved overlapping a model of BP1253 with a monomer of the type-I LOG protein of *M. marinum* co-crystallized with AMP (MmLOG, PDB 3SBX)^14,19^. BP1253 was modeled on a monomer of the type-II LOG protein of *T. thermophilus* TT1465 (TtLOG), which shares 48% of amino-acid identity with BP1253 (personal observation). The overlap between 3SBX and BP1253 produced a Root Mean Square (RMS) value of 1.21 Å, showing a great similarity between the binding pockets (Supplementary Fig. S3). Most importantly, surface plasmon resonance (SPR) confirmed experimentally the binding of AMP to BP1253 with a K_D_ of 5.7 µM. Interestingly, BP1253 also binds GMP and IMP with a K_D_ of 38.6 and 71.8 µM, respectively (Fig. 3), and TMP and UMP with K_D_ in the millimolar range (2.4 and 6.1 mM, respectively, data not shown). No binding was identified with CMP. Overall, these data show a wide binding capacity for BP1253 to purine and pyrimidine monophosphate nucleotides.

### BP1253 is a LOG with phosphoribohydrolase activity

**Figure 4:**
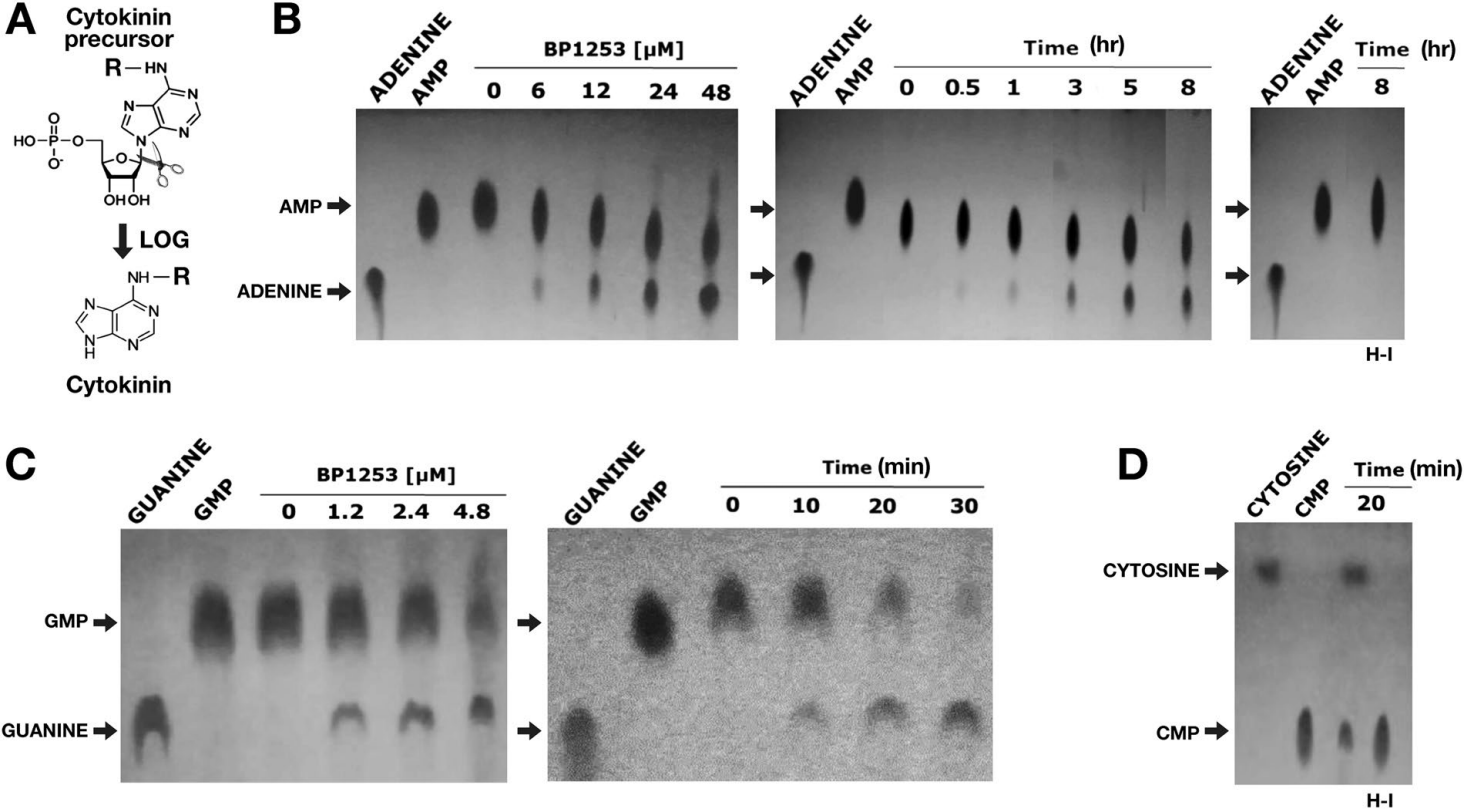
Dose-response and time-course experiments reveal phosphoribohydrolase activity of BP1253. (**A**) Schematic representation of phosphoribohydrolase (PRH) catalyzed reaction. (**B**) Experiments performed at 30 °C in the presence of 20 mM AMP as substrate. On the left, dose-response experiment carried out after 6 hours of incubation. The BP1253 concentrations used were 6, 12, 24 and 48 µM. In the center, time-course assay carried out with 24 µM of enzyme at different incubation time as shown in the figure. On the right, the TLC with the protein inactivated at 99 °C for 10 min. as negative control (H-I) after 8 hours of incubation at 30 °C. After heat inactivation of the assay mixture at 95 °C for 5 min, the products were separated by TLC with 1 M NaCl as mobile phase on Cellulose F plastic sheets. The dots were identified under UV light at 264 nm. Arrows indicate the substrate AMP and the cleaved product adenine. (**C**) Assays performed at 30 °C in the presence of 20 mM GMP. Dose-response experiment carried out in 20 minutes with 1.2, 2.4 and 4.8 µM enzymatic concentrations of BP1253 and time-course experiment performed with 2.4 µM of BP1253 at different incubation times as shown in the Figure. The reaction mixture was inactivated with 1 M NaOH. The products of the reaction were separated by TLC and dots visualized as described above. Arrows indicate the substrate GMP and the cleaved product guanine. (**D**) Assays carried out with 2.4 µM of BP1253 at 30 °C in the presence of 20 mM CMP for 20 minutes. The control reaction was performed with BP1253 heat-inactivated at 99 °C for 10 min. (H–I). The products of the enzymatic reaction were separated by TLC on Cellulose F plastic sheets with acetone/water 30/70 (v/v) with the addition of 0.2 mol/l NaCl as mobile phase, while dots were visualized as described above. Arrows indicate the substrate CMP and the cleaved product cytosine.

**Figure 5:**
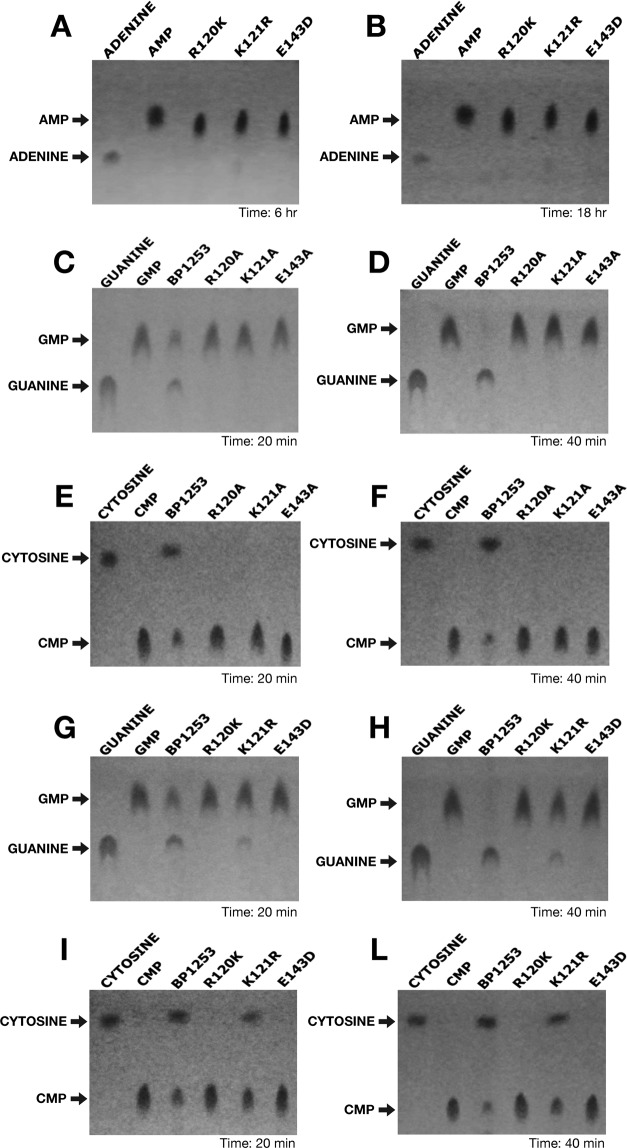
Site–directed mutagenesis identifies the catalytic residues of BpLOG. The phosphoribohydrolase activity of BpLOG variants with conservative or non-conservative mutations was quantified at 30 °C in the presence of 20 mM of the different nucleotides as substrates. BpLOG variants with conservative variations with AMP (**A**, **B**), for 6 (**A**) or 18 (**B**) hours, using GMP as substrate with non-conservative (**C**, **D**) or conservative (**G**, **H**) mutations for 20 min. (**C**, **G**) or 40 min. (**D**, **H**), BpLOG using CMP as substrate with non-conservative (**E**, **F**) or conservative (**I**, **L**) for 20 (**E**, **I**) or 40 (**F**, **L**) min. Mixtures were heat inactivated (AMP, CMP) at 95 °C for 5 min. or inactivated (GMP) with 1 M NaOH. The products were separated by TLC with 1 M NaCl as mobile phase on Cellulose F plastic sheets (AMP, GMP) or with acetone/water 30/70 (v/v) with the addition of 0.2 mol/l NaCl as mobile phase (CMP). The dots were visible under UV light at 264 nm. Arrows indicate the different substrates and the cleaved products. Experiments repeated three times gave identical results.

The high similarity in amino acid sequence and the SPR analysis led us to hypothesize that BP1253 functions as a LOG protein. However, since BP1253 was previously annotated as LDC, we first performed a specific assay for LDC activity^20^. No LDC activity was detected (data not shown) showing mis-annotation of this protein. We then quantified PRH, an activity only recently demonstrated in human pathogens^15,16^, which leads to the formation of free and active cytokinins after cleavage of the N-glycosidic linkage between the N^6^-modified adenine and ribose 5’-monophosphate (Fig. 4). The cleavage of AMP was dose- and time- dependent (Fig. 4). SPR results suggested carrying out the enzymatic assay also with GMP as substrate. As shown in Fig. 4, a ten-fold less concentrated BP1253 cleaved GMP almost completely after 30 minutes of incubation, indicating GMP as a more efficient substrate than AMP. A high PRH activity was recorded also with CMP (Fig. 4). This result is in apparent contrast with the undetectable binding of CMP to BP1253 by SPR and could be due to the rapid cleavage of CMP by BP1253. Subsequently, the involvement of R120 and E143 in enzyme catalysis and of K121 in substrate binding was confirmed by using BP1253 mutants (Supplementary Fig. S4). Substitutions of R120 and E143 with conservative residues resulted in a total absence of activity (Fig. 5), while the replacement of K with R at the residue 121 (K121R) produced a minimal hydrolysis both with CMP and GMP, not comparable to the wild type (Fig. 5). The results obtained from activity and mutagenesis studies strongly support BP1253 as being a *B. pertussis* ortholog of plant LOG and, henceforth, we will refer to it as BpLOG.

### LC-MS analysis of *B. pertussis* citokinins

**Figure 6:**
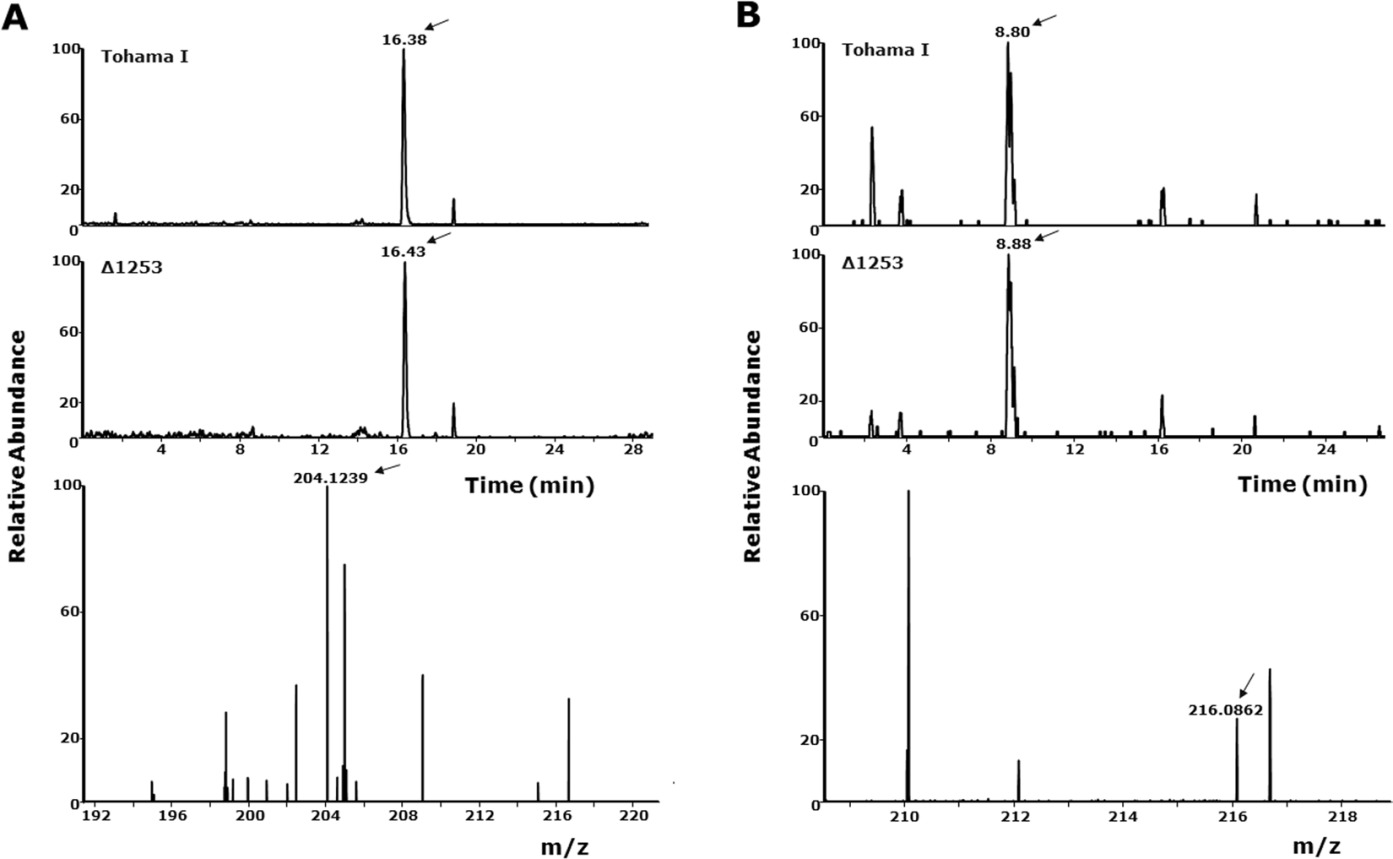
Natural occurrence of isopentenyl adenine and kinetin in lysates of Tohama I and knock-out Tohama I Δ1253 strain. Cytokinins from bacterial lysates, prepared as described in Methods, were purified, dried in SpeedVac and dissolved in 100 µl of 5% methanol in water and centrifuged as described in Methods. The supernatant was then analyzed in LC-MS. (**A**) Extracted ion chromatogram for isopentenyl adenine in sample Tohama I and knock-out Tohama I Δ1253 strain. The chromatographic peak at retention time of 16.4 min. analyzed in mass spectrometry in a positive ion mode with electrospray, resulted in the experimental elemental composition of C_10_H_14_N_5_ (−2.21 ppm) (lower panel), with a mass of 204.24 Da (arrow) that corresponded to protonated isopentenyl adenine (203.24). (**B**) Extracted ion chromatogram for kinetin in sample Tohama I and knock-out Tohama I Δ1253 strain. The chromatographic peak at retention time 8.8 min. analyzed as described above gave the experimental elemental composition of C_10_H_10_ON_5_ (−8.45 ppm) (lower panel), with a mass of 216.08 (arrow) that corresponded to protonated kinetin (215.16). Experiments were performed in triplicate with identical results. The collision energy used for the fragmentation was 26 a.u.

**Figure 7:**
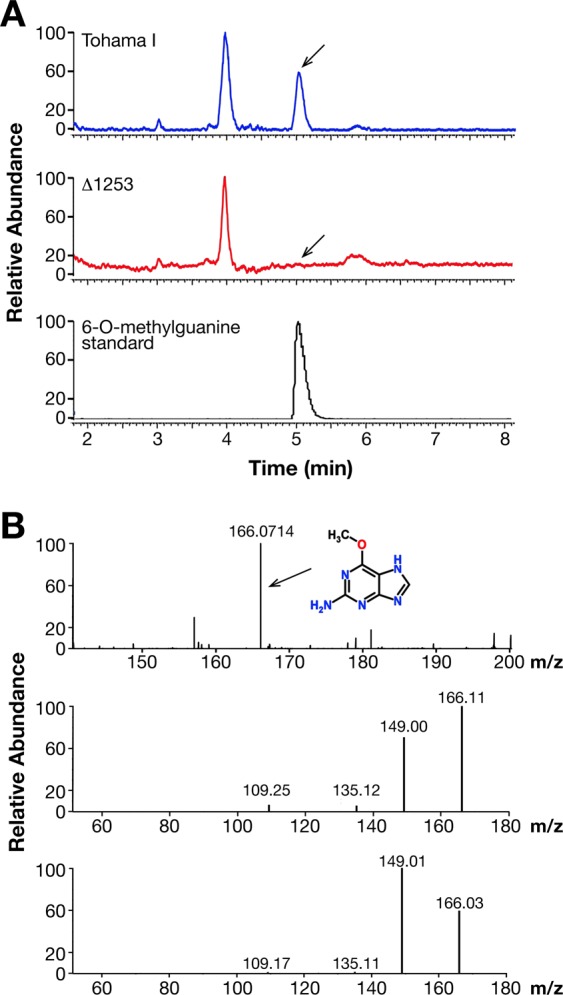
LC-MS analysis of BpLOG physiological product. The growth medium was centrifuged to eliminate bacteria and then filtered with a membrane 3 K, the volumes reduced at 4 ml using SpeedVac, acidified with acid formic and cytokinins purified with solid phase extraction with a MCX matrix. The purified cytokinins were dried in SpeedVac, dissolved in 100 µl of 5% methanol in water and centrifuged. The supernatant was then analyzed in LC-MS. (**A**) The peak seen in the Tohama I growth medium was completely absent in the medium of the knock-out Tohama I strain. This peak isolated and analyzed in mass spectrometry in a positive ion mode with electrospray resulted in a compound of 166 Da. The 6-O-methylguanine standard was run on HPLC using the same conditions used for supernatant samples and the retention time obtained was similar to the physiological product. Experiments were performed in triplicate with identical results. (**B**) The MS/MS comparison analysis between the physiological product and the 6-O-methylguanine confirmed the identity of the product synthesized by BpLOG. The collision energy used for the fragmentation was 26 a.u.

Since BpLOG is a member of the LOG family, in analogy with its homolog proteins it should be able to produce cytokinins. Cytokinins are plant hormones, which are produced in small quantities and released in the extracellular space. In order to verify the biological activity of BpLOG within the context of live bacteria, we generated a knock-out Tohama I Δ1253 strain and, using the LC-MS/MS approach, analyzed the cytokinins in the culture supernatants and lysates of either the wild-type or knock-out strain. We first investigated the presence of canonical cytokinins and identified the presence of isopentenyl adenine and kinetin in the bacterial lysates of both wild-type Tohama I and Δ1253 knock-out strain (Fig. 6). Further analyses revealed a peak with a retention time of 5 minutes in the supernatant of wild-type Tohama I, but not of Δ1253 mutant strain (Fig. 7). Identical result was also obtained with bacterial lysates (data not shown). This peak, with an apparent molecular formula of C_6_H_7_N_5_O, corresponded to a compound with a molecular weight of 165 Da, which appeared in the chromatogram with a mass of 166 Da due to the positive ion mode method used for detection. In the data bank the best fitting compound with this formula was 6-O-methylguanine. Indeed, the retention time of the synthetic 6-O-methylguanine was identical to the one isolated in Bp (Fig. 7). In addition, the fragmentation mass spectra of the isolated physiological product and of the synthetic 6-O-methylguanine were identical, showing that BpLOG synthesizes the 6-O-methylguanine as a physiological product (Fig. 7). Notably, we identified 6-O-methylguanine in bacterial lysates and in supernatants of the clinical Bp isolates B3629 and B3621^6^ (data not shown).

### BpLOG is negatively related to oxidative stress

**Figure 8:**
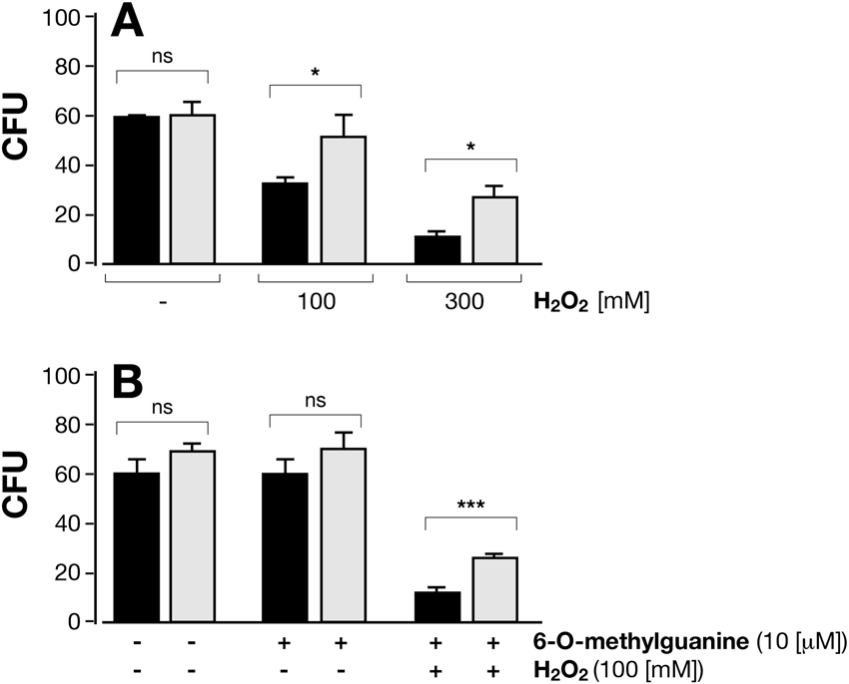
Deletion of BpLOG confers greater resistance to hydrogen peroxide. Wild-type Bp (*black columns*) and Δ1253 mutant Bp (*grey columns*), grown for 24 hours in Steiner-Scholte medium, were treated for 30 minutes with: (**A**) 100 mM or 300 mM H_2_O_2,_ or left untreated; (**B**) 10 µM 6-O-methylguanine alone or in combination with 100 mM H_2_O_2,_ or left untreated_._ At the end of the treatment, bacteria were diluted 1:10^9^, plated on Bordet-Gengou agar plates and incubated at 37 °C for three days when the number of colonies was counted (CFU). Experiments were repeated three times. Mean CFU ± SD of Δ1253 and wild-type Bp are reported. *P < 0.05; ***P < 0.001, as measured by two-tailed Student’s t-test; ns, not significantly different.

In *M. tuberculosis* the excess of cytokinin breakdown products increased sensitivity to nitric oxide (NO)^15,21–23^. Since BpLOG is a homologous of MtLOG, we tested the susceptibility of Bp to oxidative stress conditions. Wild-type Bp bacteria were more sensitive to oxidative stress conditions as compared to BpLOG-deficient mutant bacteria Δ1253 as shown by the reduced number of viable bacteria after treatment with 100 mM or 300 mM hydrogen peroxide (H_2_O_2_, Fig. 8A). These results suggested that 6-O-methylguanine produced by BpLOG sensitizes Bp to the oxidative stress due to H_2_O_2_. To test this hypothesis we incubated the two Bp strains with 100 mM H_2_O_2_ with exogenously added 10 µM 6-O-methylguanine. As shown in Fig. 8B, addition of 6-O-methylguanine together with H_2_O_2_ lowered the viability of Δ1253 knock-out strain. Importantly, addition of 6-O-methylguanine alone to either wild-type or Δ1253 mutant strain had no effect on Bp viability (Fig. 8B). Overall, these results indicated a role of BpLOG in sensitizing Bp to oxidative stress.

## Discussion

The resurgence of the pertussis infection is a serious public health problem. Waning immunity induced by the aP vaccine and lack of herd immunity can lead to Bp strains diffusion. Therefore, it is necessary to increase our basic knowledge on Bp microbiology in order to find new therapeutic/vaccine strategies and/or to improve the existing ones. In this study, combining *in silico* analyses and *in vitro* experiments, we demonstrate that BP1253 is a novel member of the LOG family since it contains the highly conserved LOG motif PGGxGTxxE, the catalytic amino acids Arg and Glu, and possesses PRH activity. Through this activity, LOG proteins synthesize cytokinins. Although BpLOG has a primary sequence similar to hexameric type-II LOGs, the dimeric state and the presence of specific amino-acids strongly support the hypothesis that BpLOG could represent a novel class of type-II LOGs. Noticeable differences are the presence of Asp at position 142 occupied by Glu in the majority of type-I LOGs, and Phe at position 117. Another noteworthy difference is at position 118, which is occupied by Tyr in BpLOG and by Phe and His in the other LOGs. The presence of different residues all involved in the formation of the prenyl-group binding site could explain the capability of this protein to bind and hydrolyze more effectively molecules like GMP and CMP. These peculiarities that make BpLOG unique, are mirrored by the production of the physiological product. In fact, BpLOG produces 6-O-methylguanine, a guanine-derivative with a methyl-group at O^6^, while the cytokinins described so far are adenine-derivatives modified at N^6^. Indeed, synthetic O^6^-substituted guanine derivatives have been described as acting like cytokinins, inducing cell division in plants^24^. Remarkably, the synthesis of the 6-O-methylguanine is in agreement with the higher PRH activity of BpLOG with GMP. The flexibility of BpLOG towards other nucleotides suggests that BpLOG could synthesize also other non-canonical cytokinins containing guanine or cytosine as nitrogenous bases. The production of modified adenine cytokinins by Bp is probably due to the presence of BP0547, which shares an amino-acid identity of 54% with the characterized type-I *P. aeruginosa* LOG^18^ and 39% with MtLOG, which are both able to synthesize adenine-derivate cytokinins^15^, but only 23% with BpLOG. While prediction studies indicate that BP0547 could be localized in the cytoplasm, BpLOG could be located in the periplasmic space or on the external membrane due to the presence of a putative trans-membrane helix (Fig. S5). The different cellular localization of the two enzymes could partially explain the low amount of 6-O-methylguanine found in bacterial lysates (not shown), while the inability to detect adenine-derivate cytokinins in the supernatant of wild-type Bp was probably due to the low amount secreted. Our results show that BpLOG is responsible for the synthesis of 6-O-methylguanine while they do not exclude that BpLOG is involved in the biochemical pathway of canonical cytokinins. This discovery has a strong impact, since 6-O-methylguanine could represent a novel class of molecules with cytokinin activity.

The capability of BpLOG to recognize and metabolize several nucleotides prompts the hypothesis that BpLOG could protect Bp by degrading non-canonical nucleotides present in the external environment, which can interfere with the physiology of the bacterium. Indeed, in *S. cerevisiae* the over-expression of the analog *LOG1* (*YJL055W*) gene conferred resistance to the chemotherapeutic drugs 6-N-hydroxylaminopurine (HAP) and 5-fluorouracil (5-FU), due to the detoxification properties of the LOG protein encoded by this gene^25^.

The function of cytokinins in Bp and in other human pathogens has just started to emerge. In plants, cytokinins are hormones that act locally or distant from the cell at very low concentrations^26^, modulating plant metabolism and morphogenesis in response to environmental factors^27^. 6-O-methylguanine could be a signaling molecule for inter-bacteria communication and/or a way to modulate the expression of specific genes helping the bacterium to establish a successful infection. Recently, it was reported that in *M. tuberculosis* isopentenyl adenine regulates the expression of genes associated with the bacterial envelope and virulence^28^. The enhancing effects of 6-O-methylguanine in combination with ROS, produced by lung epithelial cells^29^ or by resident microbes^30^, could produce compounds toxic for epithelial cells to enhance colonization and/or favor Bp towards other bacterial species competing for the same niche. Indeed, Bp could be protected from the bacterial clearance associated with an increased level of ROS^29^ by the cytokinin kinetin produced by BP0547, which reduces generation of ROS and induces a raise in the activity of the antioxidant protection systems^31–34^. The latter confirms a relation of LOGs and their products with oxidative stress in agreement with *M. tuberculosis*, where the over expression of MtLOG led to an accumulation of cytokinin degradation products which, interacting with NO, are toxic for the bacteria^15^. However, further investigation is required to clarify the *in vivo* role of BpLOG.

Overall, we characterized BP1253 as a LOG of the human-exclusive pathogen Bp with peculiar features. The discovery of this new LOG in Bp suggests that LOGs may play an important role in the pathogenesis of human bacterial diseases. Furthermore, a better understanding of the role of LOGs in bacterial infections could lead to the rational design of novel therapeutic strategies that are urgently needed due to the emerging concerns about antibiotic resistance.

## Methods

### Materials

Isopropyl-1-thio-β-D-galactopyranoside was obtained from Calbiochem; the BCA reagent for protein quantification and the ECL immunoblotting detection system were from Bio-Rad, the protein inhibitor cocktail from Roche, and standard Bovine Serum Albumin (BSA) was obtained from Pierce. SimplyBlue SafeStain, Nu-PAGE gels and iBlot membranes were purchased from Invitrogen. All other reagents used in this study were from Sigma Aldrich.

### Bioinformatics analysis

The BLAST algorithm was used to investigate the sequence homologies between BP1253 and LOGs, while *Clustal omega* was used to realize amino acid sequence alignments with specific type-I and type-II LOGs. The structural analysis was performed by means of PDB Viewer and to create the figure and represent the sequence alignment *ESPript* 3.0 software was used. The graphic software PyMOL was used to visualize the overlaps, while the phylogenetic tree was generated with MAFFT and visualized through the software tool Archaeopteryx.js.

### Bacterial strains and growth conditions

The following *B. pertussis* strains were used in this study: Tohama I-derivative BP536^35^, the clinical isolates BP3629 and BP3621^6^. Bacteria were stored at −80 °C and recovered by plating on Bordet-Gengou (BG) agar plates, supplemented with 15% (v/v) sheep blood for 3 days at 37 °C. The bacteria were then inoculated at an initial 600 nm optical density (A_600_) of 0.05–0.1 in Stainer-Scholte medium supplemented with 0.4% (w/v) L-cysteine monohydrochloride, 0.1% (w/v) FeSO_4_, 0.2% (w/v) ascorbic acid, 0.04% (w/v) nicotinic acid, and 1% (w/v) reduced glutathione. Cultures were grown in rotary shakers at 37 °C.

### Generation of *E. coli* expressing recombinant BP1253 protein and purification procedure

The synthetic *gene1253* from Bp pertussis was synthesized and assembled by GeneArt (Thermofisher) starting from synthetic oligonucleotides and/or PCR products. The fragment was inserted into pET24b(+)_D455 as a fusion construct with a carboxyl-terminal 6x histidine tag separated from the last amino acid of the protein by a linker of 2 amino-acid residues. The sequence congruence verified by sequencing of the final construct within the insertion sites was 100%. *E. coli* strain BL21(DE3) cells (England BioLabs) were transformed with the above constructs and used for protein expression. Cells were grown using BioSilta medium (Enpresso B Animal-free growth systems), at 30 °C for 8 h with gentle shaking (160 rpm). The expression of *Bp1253* gene was induced to an A_600_ of 0.5–0.6 by the addition of 1 mM isopropyl β-D-1-thiogalactopyranoside (IPTG) 24 h at 17 °C. Cells were harvested by centrifugation and re-suspended in lysis buffer containing 50 mM NaH_2_PO_4_, 300 mM NaCl, pH 7.4 and EDTA-free protease inhibitor (Boheringer Mannheim) at a ratio of 10 ml of lysis buffer per 1 g of bacterial pellet. After lysis, performed via sonication (Qsonica Q700), cell lysates were clarified by centrifugation at 15000 x g for 50 min at 4 °C, and the supernatant, containing the expressed protein, was filtered using 0.22 μm membrane filters (EMD Millipore filters) before starting the first chromatography step. BP1253 was purified by Co^2+^-affinity chromatography (5 mL HiTrap TALON crude, GE Healthcare) at 25 °C using an AKTA purifier 100 system (GE Healthcare). The column was equilibrated with buffer A (50 mM Tris pH 8, 300 mM NaCl). After loading the crude extract, the column was washed with 10 bed volumes of buffer A. Bound proteins were eluted with buffer A containing 500 mM imidazole. The content of lipopolysaccharide (LPS) on the purified protein was checked using the Endosafe nexgen-PTS system (Charles River). When the content of LPS was out of the range, it was removed using the EndoTrap Red columns (Hyglos). The purity of the protein was checked using 4–12% SDS–PAGE gradient gels in MES buffer, after identification of the fractions containing BP1253 samples were pooled and stored at −20 °C for subsequent analysis.

### Size-exclusion chromatographic analysis

The investigation of BP1253 oligomerization was performed using analytical size exclusion chromatography. The chromatographic step was performed using a BEH200 column 4.6 × 300 mm (Waters) at a flow of 0.4 ml/min with a buffer containing 10 mM NaH_2_PO_4_ and 400 mM (NH_4_)_2_SO_4_ pH 6.0. Protein samples of 15 μl, 0.62 mg/ml and 3 mg/ml were analyzed. The molecular weights of the different forms of BP1253 were calculated from a calibration curve based on standard proteins.

### Generation of the BP1253 Knockout strain

The deletion strain for the genes BP1251–1252–1253 of the *B. pertussis* BP536 Tohama I-derivative was constructed as follows. The 5′ and 3′ extremities of the locus were amplified by PCR using Bp chromosomal DNA as template and the oligonucleotides

FlankingUP-Fw ccgGAATTCCGAAAACCGTAGCGGTCGAA and

FlankingUP-rev ggaGGATCCGGACCGATGTCGGCCAATTT,

FlankingDOWN-Fw ggaGGATCCCGCGTCTATGTCGACCACG and

FlankingDOWN-rev cccAAGCTTCGAACTGCACCTGACCATCC as primers, respectively.

The amplicons were then successively introduced as EcoRI-BamHI and BamHI-HindIII fragments into pUC19, together with a BamHI-BamHI fragment encoding the kanamycin resistance cassette for selection. The resulting EcoRI-HindIII fragment was then purified and introduced into the EcoRI-HindIII sites of pSORTP1, a mobilizable suicide plasmid used for conjugation between Bp and *E. coli*. Conjugation was performed on BG-blood agar plates containing 10 mg/ml MgCl_2_ for 5 hours, and co-integrates were selected on BG-blood agar plates containing 10 µg/ml gentamycin and 20 µg/ml nalidixic acid to prevent growth of the *E. coli* donor. Single crossing overs determine the insertion of the pSORTP1 vector in the chromosome and confer gentamycin resistance and streptomycin sensitivity. Double crossing overs were selected by a successive step on BH-blood plates containing 25 µg/ml kanamycin and 400 µg/ml streptomycin. After 3 to 4 days growth on selective media, isolated kanamycin and streptomycin-resistant colonies were analyzed by PCR to confirm the deletion.

### High-Throughput purification of BP1253 mutants

Single point conservative and non-conservative mutations were inserted into the *Bp1253* gene to generate six different mutants. Mutated genes were synthesized and assembled by GeneArt (Thermofisher) starting from synthetic oligonucleotides and/or PCR products, using the same plasmid and procedure as described above. The mutants generated were R120A, R120K, K121A, K121R, E143A, and E143D. Purification of mutants was performed under vacuum conditions in a 96-well Vacuum plate. Cells were lysed, using B-PER (Sigma) and applied on a His Multitrap HP 50 ml NiSepharose High Performance 96 wells previously washed with water and equilibration buffer (300 mM NaCl, 50 mM NaH_2_PO_4_, pH 8). After loading the samples, the plate was washed with 80 vol. of washing buffer (300 mM NaCl, 50 mM NaH_2_PO_4_, 20 mM imidazole, pH 8) at 25 °C. The His-fusion proteins were eluted in two steps by addition of 6 vol. of elution buffer (300 mM NaCl, 50 mM NaH_2_PO_4_, 500 mM imidazole, pH 8) twice. All the elutions related to the same protein were subsequently pooled. All purification steps were carried out applying a vacuum not exceeding the maximum pressure of 5 mmHg.

### SDS-PAGE and western blot

SDS-PAGE and western blot (WB) analyses were performed to monitor expression, purity and identity of BP1253 during purification. Samples mixed with 1X NuPage LDS loading buffer and 1X NuPage Sample reducing agent (Life Technologies) were heated at 70 °C for 10 min. before loading 20 μl of the mixture onto a 4–12% gradient NuPAGE Bis-Tris gel (Life Technologies). SeeBlue Plus Prestained Standard markers were run on each gel, which was stained with Comassie Blue to visualize the proteins. Separated proteins were electro-transferred onto nitrocellulose membranes with iBlot 2 Dry Blotting System (Life Technologies). The membranes were blocked for 60 min with 0.1% Tween 20 and 10% milk in phosphate buffer solution (PBS), and then incubated for 1 h with specific mouse polyclonal α-BP1253 antibodies (1:500 dilution) in 0.1% Tween 20 and 3% milk buffer in PBS. After three washes with 0.1% Tween 20 in PBS (T-PBS) the membrane was incubated with a secondary rabbit α-mouse horseradish peroxidase conjugated antibody (Jakson Immune research Laboratory, 1:1000 dilution) for 30 min at room temperature. Bound antibodies were visualized, after washing membranes three times with T-PBS, using the ECL immunoblotting detection system (Bio-Rad) according to the manufacturer’s instructions.

### Phosphoribohydrolase activity assay

Phosphoribohydrolase activity was assessed by detecting the adenine ring compounds, after separation by thin layer chromatography (TLC) as described^36^. Briefly, enzyme reaction was carried out in a mixture containing, in a final volume of 20 μl, 40 mM Tris-HCl (pH 8), 20 mM monophosphate nucleotide as substrate and the amount of purified recombinant protein as specified in figure legends. After incubation at 30 °C, samples (5 μl) were denatured at 95 °C for 5 min and then 1 μl dotted on PEI-cellulose-F-plastic TLC sheet (Merck Millipore). Since the solubility of guanine is poor in water and to enhance its visibility on TLC, inactivation was performed with 1 M NaOH (v/v). The nitrogenous base ring was separated from the phosphoribose moiety using a TLC method with a mobile phase containing 1 M sodium chloride, except for pyrimidine rings where the mobile phase was acetone/water 30/70 (v/v) with the addition of 0.2 mol/l NaCl. After the run the sheet was completely dried and the separated dots visualized by means of a UV lamp (264 nm).

### Generation of mouse immune sera

BALB/c mice (10 female/group, 6-week old) (Charles River Laboratories International Inc.,

Wilmington, MA) received three intraperitoneal immunizations, with a 4-week interval, with aluminum hydroxide (2 mg/ml) adjuvanted recombinant BP1253-His (10 μg per dose) at one fifth of a human dose. Sera were collected before immunization and 2 weeks after the third immunization. Control mice immunized with adjuvant only were included in the experiments. All the experiments involving animals were performed in compliance with Italian law, with the approval of the local Animal Welfare Body (AWB 2014/06) followed by authorization of Italian Ministry of Health.

### Identification and characterization of cytokinins by LC-MS/MS

For the analysis of the cytokinins, the supernatant was obtained by spinning down the bacterial growth broth at 9000 x g for 20 min. and filtered and treated as described^37^. Briefly, the supernatant was cleared by molecules with MW higher than 3 kDa using a protein concentrator 3 K (Amicon), following the vendor’s instructions. Subsequently, the samples were concentrated down to 1 ml volume through a Speed Vac and loaded on a solid phase extraction (SPE) column (MLX matrix, Oasis). Before loading, samples were previously acidified to pH 3 with 98% formic acid. The column was then washed with 0.5 mL SPE load solvent (1 M formic acid), followed by 1 mL water. The samples of interest were eluted with 0.5 mL of freshly prepared solvent 2 (0.35 M ammonium hydroxide in 70% methanol, to 70 mL methanol there was the addition of 2.5 mL of 25% ammonium hydroxide filled to 100 mL with distilled water), the flow-through collected into a new 2 mL microcentrifuge tube was dried in SpeedVac at 10 mBar and 40 °C. The dried fractions were stored at −20 °C until LC-MS analysis. At the time of starting the analysis in LC-MS the dried samples were dissolved in 100 μL 5% methanol in water and centrifuged at 20000 × g, 4 °C for 20 min. The supernatant was transferred into an autosampler vial and used for the analysis without further modification. LC-MS/MS analysis was performed using an LTQ-Orbitrap XL coupled with an Ultimate 3000 HPLC system equipped with a reverse phase column Luna C18(2) 100 mm × 2 mm, 3 µm, 100 Å. The mobile phases A and B used for the analysis of the sample were 0.1% formic acid in water and 0.1% formic acid in acetonitrile respectively. The gradient started with 100% of A, maintained for 3 minutes and the phase B increased up to 20% in 12 minutes. Then after two minutes the phase B reached 100% and it was held constant for two minutes. The flow rate was set to 200 µl/min. UV at 268 nm while a positive ion mode with electro spray was used for mass spectrometric detection. The ESI ion source spray voltage was set to 5 kV, the capillary temperature was 300 °C, and tube lens and capillary voltage were 110 and 35 V respectively. The sheath and auxiliary gas (N_2_) were set to 20 and 5 a.u. The mass spectra and the MS/MS spectra were recorded using the data dependent scan mode at resolution of 30000, while the fragmentation mass spectra were recorded in low resolution with a collision energy of 26 a.u.

### Surface plasmon resonance experiments

Surface plasmon resonance (SPR) experiments were performed using a Biacore T200 instrument (GE Healthcare). The recombinant BP1253 protein was amine-coupled at 20 µg/mL in 50 mM NaOAc at pH 4.5 to the surface of a CM5 sensor chip (GE-Healthcare) and activated with 50 mM 1-ethyl-3-(3-dimethylaminopropyl)-carbodiimide (EDC)/50 mM N-hydroxysuccinimide (NHS). Typically, response levels of 8000–9000 RU were achieved. After priming the system with HBS-EP + buffer (10 mM HEPES, 150 mM NaCl; 3 mM EDTA; 0.05% Surfactant P20; pH 7.4), affinity data were generated under steady state conditions by injecting ligand nucleotides dissolved in HBS-EP + at 30 µl/s and 25 °C for 60 s, then allowed to dissociate for another 60 s. Blank-subtracted sensorgrams were analyzed using the Biacore T200 Evaluation Software v. 3.0. Response values were evaluated 4 sec before the end of each injection and averaged over a window of 5 sec.

### Phenotypic characterization

Bacteria stored with glycerol were inoculated at initial 600 nm optical density (A_600_) of 0.05–0.1 in Stainer-Scholte medium supplemented with 0.4% (w/v) L-cysteine monohydrochloride, 0.1% (w/v) FeSO_4_, 0.2% (w/v) ascorbic acid, 0.04% (w/v) nicotinic acid and 1% (w/v) and reduced glutathione. Cultures were grown in rotary shakers at 37 °C for 24 h. Subsequently, we tested the bacterial susceptibility to oxidative stress by treating cultures with 100 and 300 mM H_2_O_2_ (Sigma) for 30 min. as specified in the Figure legend. The viability of the *Bp* strains was measured after treatment.

### Multi angle light scattering

Multi-angle light scattering (MALS) analysis was performed in an HELEOS (WYATT Technology) in combination with a SEC separation. SEC was carried out with Sepax Zenix SEC-300 3 mm 7.8 × 300 mm at a flow of 0.5 ml/min with PBS as running buffer. The BP1253 for the analysis was diluted in PBS buffer at a final concentration of 0.6 mg/ml. Results were analyzed using the ASTRA software 3.1.

### Statistical analysis

Data are presented as the means ± SD and the two-tailed Student’s t-test was used to analyze significance of data. Values of *P* <0.05 were considered and referred to as significant.

### Protein assay

Protein concentration was measured with the bicinchoninic acid (BCA) protein assay^38^ (Thermo Scientific-Pierce, Rockford, IL, USA), using BSA (2 mg/ml) as a standard, equal amounts of proteins were resolved by SDS-PAGE.

### Ethics statement

All procedures involving animals were approved by the Animal Care and Ethical Committee of GSK Vaccines and the experiments were performed in compliance with Italian law, with the approval of the local Animal Welfare Body (AWB 2014/06) followed by authorization of the Italian Ministry of Health.

## Supplementary information

Supplementary Information
