# Supplementary material for: A LONELY GUY protein of Bordetella pertussis with unique features is related to oxidative stress
Source: Sci Rep. 2019 Nov 19;9:17016. doi: 10.1038/s41598-019-53171-9 (PMC6864091; doi:10.1038/s41598-019-53171-9)
Supplement: Supplementary file 1 — Supplementary Information [file 41598_2019_53171_MOESM1_ESM.docx]

**A LONELY GUY protein of *Bordetella pertussis* with unique features is related to oxidative stress**

Filippo Moramarco, Alfredo Pezzicoli, Laura Salvini, Rosanna Leuzzi, Werner Pansegrau and Enrico Balducci

**Supplementary Information**

This Supplementary Information contains 5 Supplementary figures

**
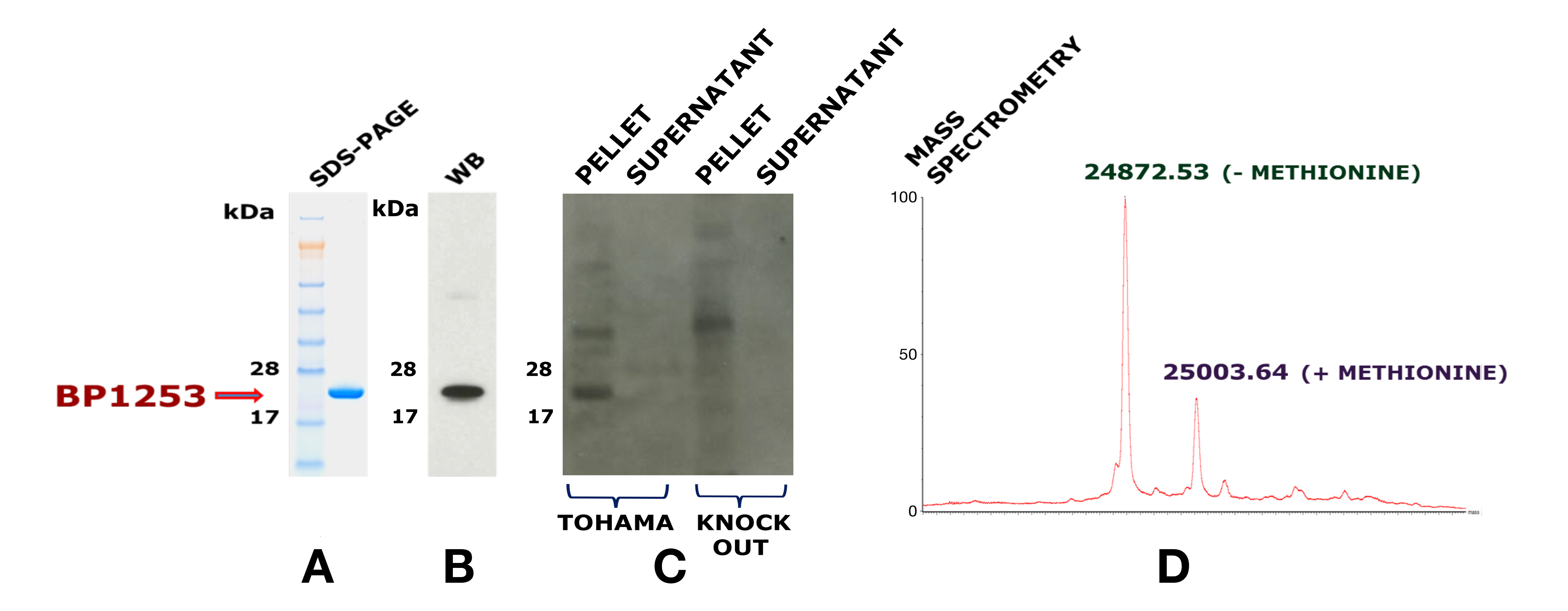
**

**Figure S1 Expression, purification and BP1253 identification.** BP1253 was cloned in *E. coli* BL21 (DE3) and purified after IPTG induction trough HiTrap TALON crude. (A) The eluate, 2 µg, was evaluated by SDS-PAGE in 4-12% gradient NuPAGE Bis-Tris gel run with 2-(N-morpholino)ethanesulfonic acid (MES) and stained with Coomassie Blue. Molecular mass markers (kDa) are indicated on the left. (B) The identity of the purified protein was further confirmed by immunoblot. The experiment was performed with 25 ng of BP1253 and with mouse polyclonal α-BP1253 (1:500 dilution) as described in Methods. Molecular weights of proteins markers are shown on the left. (C) Expression of BpLOG in bacterial lysates and supernatants of Tohama I and knock-out Tohama I Δ1253 strain. Bacterial lysates were prepared as described in Methods. The amount corresponding to 1 OD of each strain was dissolved in 100 μl of loading buffer with reducing agent and heated at 70°C. From these, 30 μl were loaded onto the gel. The corresponding supernatants, after TCA precipitation, were dissolved in 20 μl of loading buffer with reducing agent, heat inactivated and loaded onto the gel. The experiment was performed with mouse polyclonal α-BP1253 (1:500), equal loading of proteins was checked by Ponceau red staining of the blot. (D) Intact mass measurement revealed a BP1253-His molecular weight of 24872.53 Da. The weight of 25003.64 Da refers to BP1253-His proteins with unremoved methionine.


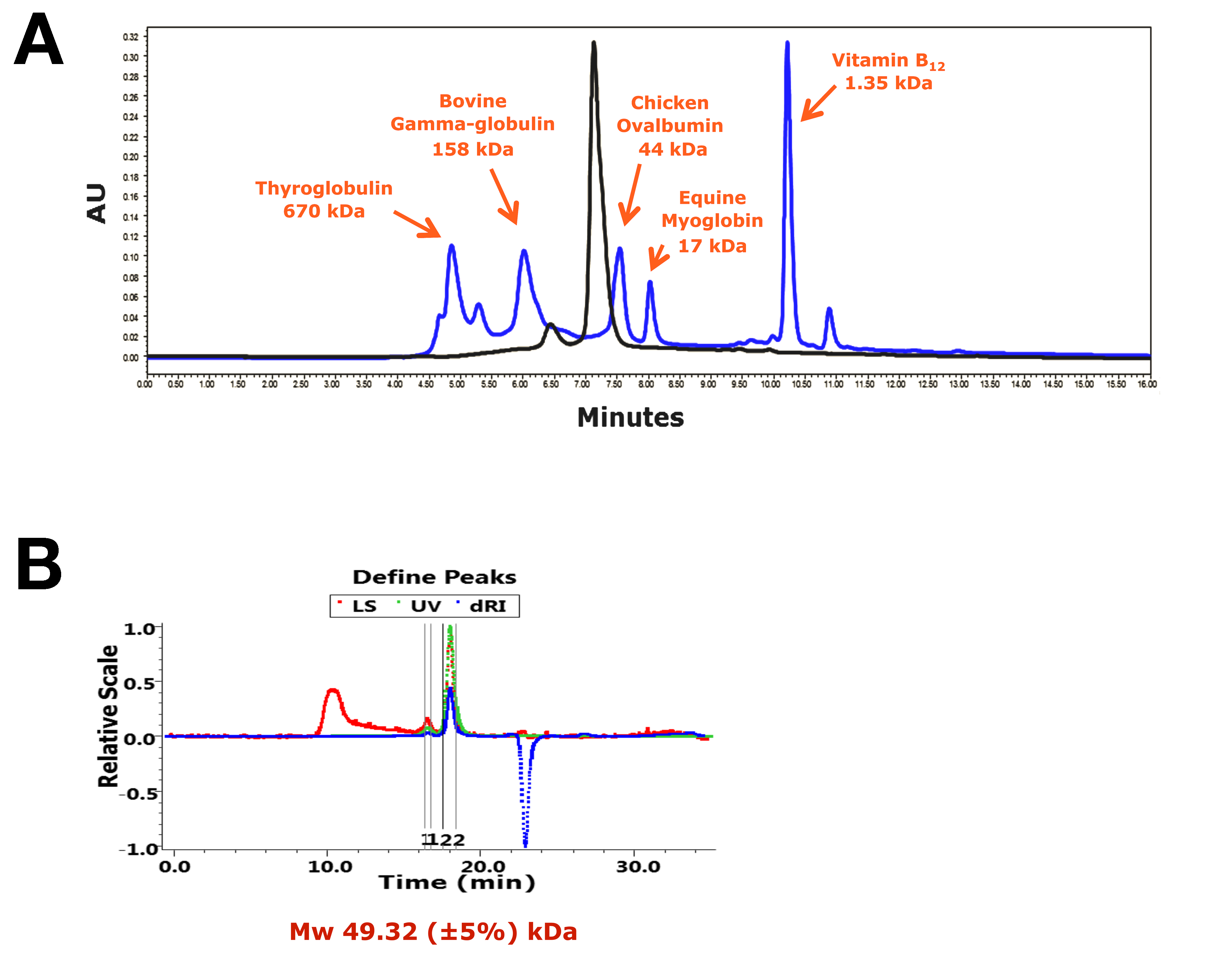


**Figure S2. SEC and Multi-Angle Light Scattering confirms the dimeric state of BP1253.** (A) Size-exclusion chromatography shows the purity of the purification and BP1253 eluting as a dimer with a molecular weight of 50 kDa. The analysis was carried out with concentrations of 0.6 mg/ml and 3 mg/ml of protein. The peak corresponding to BP1253 is marked in black, while the proteins standards with their own molecular weights are highlighted in blue. The figure refers to 3 mg/ml protein concentration. (B) MALS analysis confirmed the dimer as the oligomeric state of BP1253. The study was performed in combination with SEC using a final protein concentration of 0.6 mg/ml. The data were analyzed with ASTRA software 3.1.


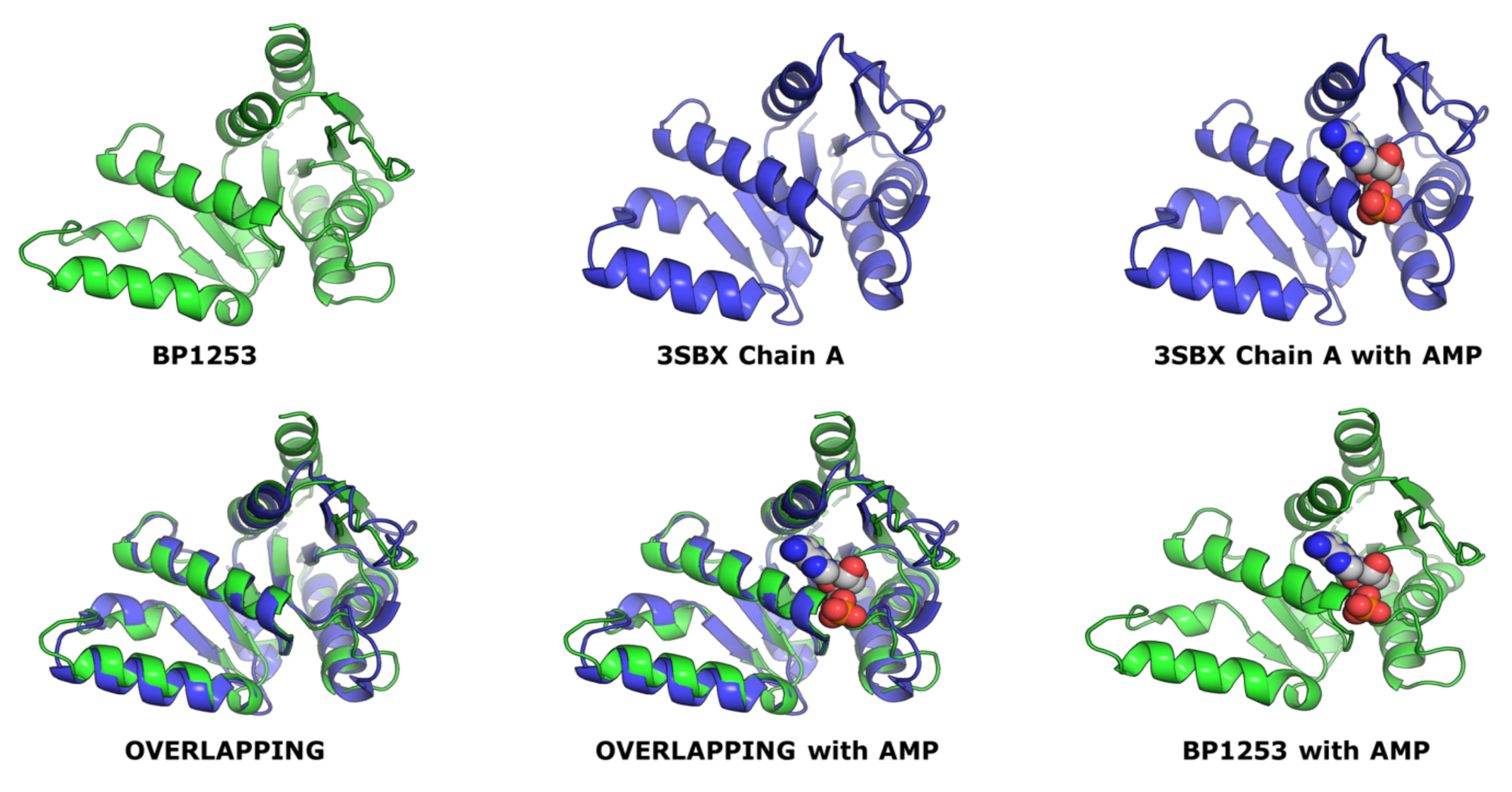


**Figure S3. Overlapping between monomer structures of MmLOG and a modeling of BP1253.** The prediction data obtained from the overlapping gave a RMS value of 1.21 Å and showed the analogy of the two binding sites. The modeling was created through PDB Viewer on the base of 48% sequence homology among BP1253 and *T. thermophilus* TT1465 protein, TtLOGII. 3SBX represent the PDB ID of TtLOGII, while chain A indicates a monomer of the crystallized protein. The overlapping was realized with PDB Viewer and the figures of single monomers and overlaps were created using the graphic software PyMOL.


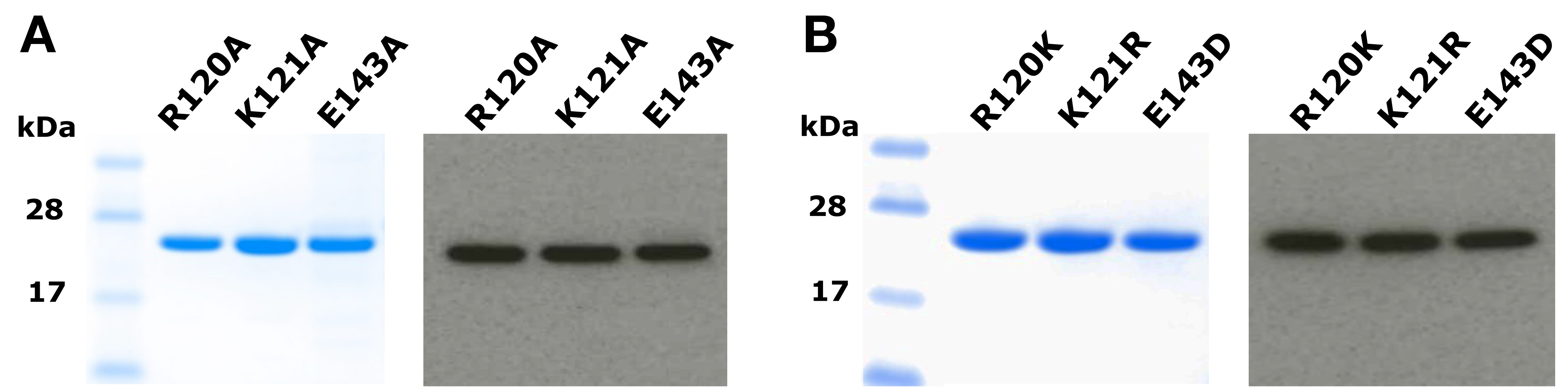


**Figure S4. Purification profile of BP1253 mutants.** BP1253 variants with specific site mutations with non-conservative and conservative amino acids were cloned in *E. coli* BL21 (DE3) and purified in high-throughput through a His Multitrap HP 96-well Vacuum plate after IPTG induction. (A) SDS-PAGE (2 µg of proteins) and Immunoblotting (20 ng of proteins) performed on R120A, K121A and E143A. Mouse polyclonal α-BP1253 (1:500) was used as the primary antibody as described in Methods. Molecular weights of proteins markers are shown on the left. (B) SDS-PAGE (2 µg of proteins) and Immunoblotting (20 ng of proteins) carried out on R120K, K121R and E143D. Molecular mass markers (kDa) are indicated on the left and the mouse polyclonal α-BP1253 (1:500) was used for the WB analysis.


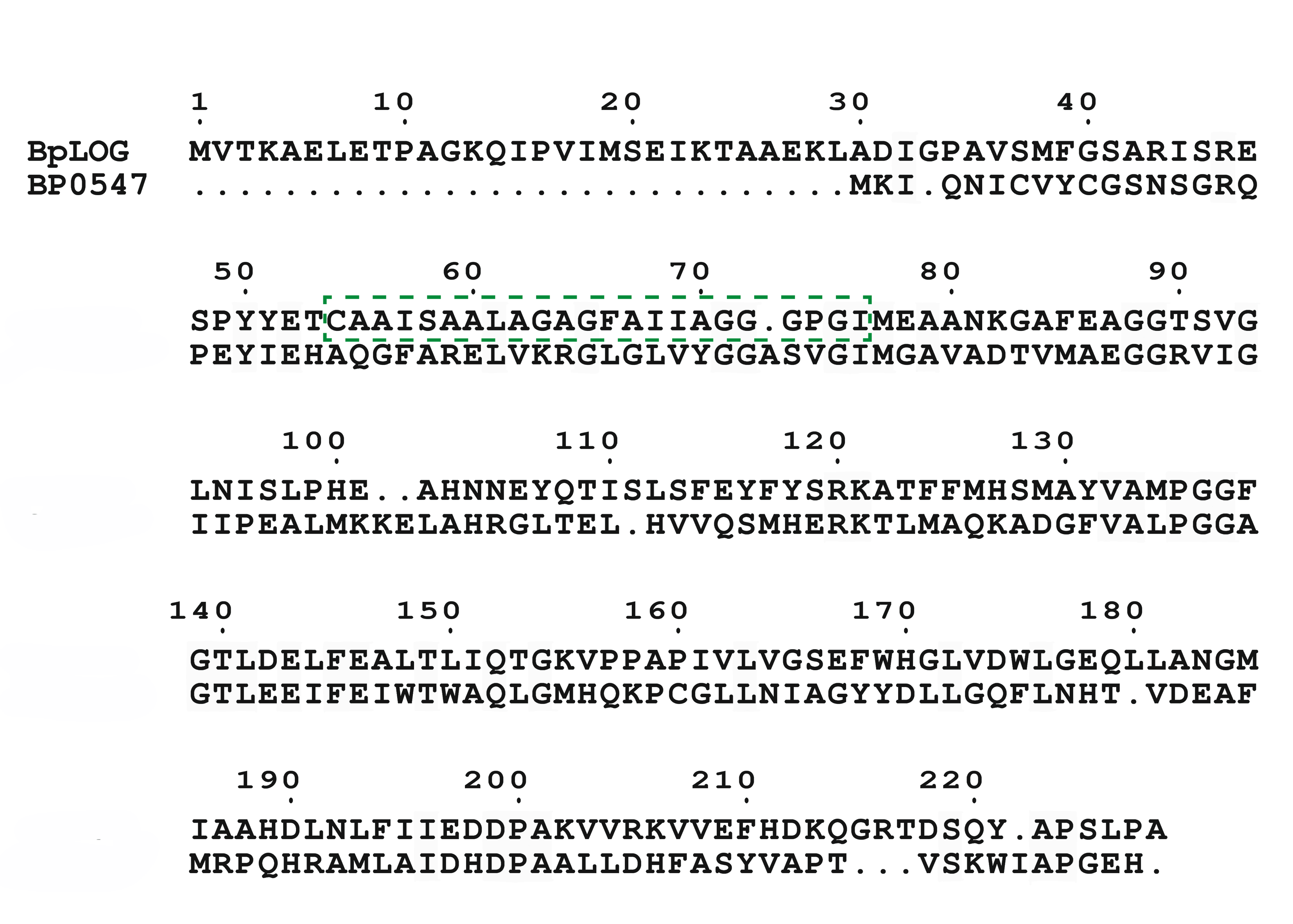


**Figure S5. Sequence alignment of BpLOG and BP0547.** The residues potentially involved in a TMhelix, predicted by TMHMM Server and indicated in a green–colored dotted rectangle in BpLOG are absent in BP0547. This alignment was generated by Clustal Omega and drawn with ESPript 3.
